# HLA class II alleles and haplotypes associated with susceptibility to type 1 diabetes and to type 1 diabetes with concomitant autoimmune diseases: a cross-sectional study from Eastern Croatia

**DOI:** 10.3389/fendo.2026.1928474

**Published:** 2026-08-05

**Authors:** Ivan Lekić, Saška Marczi, Mirjana Suver Stević, Nenad Nešković, Stana Tokić, Romana Marušić, Anđelka Bugarin, Martin Petrek, Tatjana Bačun

**Affiliations:** 1 Department of Internal Medicine and History of Medicine, Faculty of Medicine Osijek, Josip Juraj Strossmayer University of Osijek, Osijek, Croatia; 2 Department of Internal Medicine, University Hospital Center Sestre Milosrdnice, Zagreb, Croatia; 3 Department of Internal Medicine, Health Center Zagreb Centar, Zagreb, Croatia; 4 Department of Human leukocyte antigen (HLA) Typing and Genomic Diagnostics, Clinical Institute of Transfusion Medicine, University Hospital Center Osijek, Osijek, Croatia; 5 Department of Medicinal Chemistry, Biochemistry and Clinical Chemistry, Faculty of Medicine Osijek, Josip Juraj Strossmayer University of Osijek, Osijek, Croatia; 6 Faculty of Medicine, Josip Juraj Strossmayer University of Osijek, Osijek, Croatia; 7 Department of Biology, Josip Juraj Strossmayer University of Osijek, Osijek, Croatia; 8 International Medical Center Priora, Čepin, Croatia; 9 Department of Anesthesiology and Intensive Care Medicine, Faculty of Medicine Osijek, Josip Juraj Strossmayer University of Osijek, Osijek, Croatia; 10 Department of Laboratory Medicine and Pharmacy, Faculty of Medicine Osijek, Josip Juraj Strossmayer University of Osijek, Osijek, Croatia; 11 Department of Internal Medicine, National Memorial Hospital “Dr. Juraj Njavro”, Vukovar, Croatia; 12 Department of Pathological Physiology, Faculty of Medicine and Dentistry, Palacký University Olomouc, Olomouc, Czechia; 13 Department of Clinical Medicine, Faculty of Dental Medicine and Health Osijek, Josip Juraj Strossmayer University of Osijek, Osijek, Croatia; 14 Department of Endocrinology and Diabetology, Clinic of Internal Medicine, University Hospital Center Osijek, Osijek, Croatia; 15 Department of Internal Medicine, University Hospital Centre Osijek, Osijek, Croatia

**Keywords:** alleles, celiac disease, diabetes mellitus type 1, haplotypes, histocompatibility antigens class II, polyendocrinopathies, autoimmune thyroiditis

## Abstract

**Background:**

Type 1 diabetes mellitus (T1D) is an autoimmune disease with a strong genetic predisposition, largely conferred by human leukocyte antigen (HLA) class II genes. Patients with T1D frequently develop additional autoimmune diseases (AIDs). However, data on HLA class II alleles and haplotypes associated with concomitant autoimmune diseases in T1D remain limited, particularly in Central European populations. This study aimed to investigate the distribution of HLA class II allele variants and haplotypes in patients with T1D with and without concomitant autoimmune diseases affecting the thyroid, gastrointestinal tract, and skin.

**Methods:**

This cross-sectional study included 149 T1D patients who underwent low- to intermediate-resolution HLA typing of *HLA-DRB1, -DQA1* and *-DQB1* loci at the Department of HLA Typing and Genomic Diagnostics, University Hospital Centre Osijek, Croatia, between 2009 and 2026. Clinical data on associated autoimmune diseases were retrieved from electronic medical records. Previously published HLA class-II typing data of 111 unrelated blood donors from eastern Croatia were used as the control group.

**Results:**

Among 149 T1D patients, 55 (36.9%) had a concomitant autoimmune disease, most commonly affecting the thyroid (23.5%), gastrointestinal tract (7.4%), or skin (6.0%). The HLA-DRB1*03 allele variant and HLA-DQA1*05-DQB1*02-DRB1*03 (DR3-DQ2) haplotype showed the strongest association of T1D (OR 5.15 and OR 5.52, respectively; p < 0.001), whereas HLA-DRB1*04 allele variant and HLA-DQA1*03-DQB1*03(DQ8)-DRB1*04 (DR4-DQ8) haplotype showed the strongest association of T1D with concomitant autoimmune disease (OR 4.86 and OR 5.91, respectively; p < 0.001). No significant differences in HLA allele or haplotype frequencies were observed between patients with T1D alone and those with T1D and concomitant autoimmune disease.

**Conclusion:**

These findings confirm the established role of the HLA-DR3-DQ2 and HLA-DR4-DQ8 haplotypes in genetic susceptibility to T1D in a Central European population. Because a direct comparison between the T1D-alone and T1D+AID groups did not reveal significant differences, DR4-DQ8 should be interpreted as being associated with an increased risk of both phenotypes relative to healthy controls, rather than as specifically distinguishing patients with autoimmune comorbidities from those without. Given the small number of patients within individual comorbidity subgroups, disease-specific HLA associations could not be evaluated separately. Independent replication in larger, multicenter cohorts with high-resolution HLA typing is needed before these findings can be applied in clinical practice.

## 1 Introduction

Type 1 diabetes mellitus (T1D), one of the most common chronic diseases of childhood, is caused by autoimmune destruction of the pancreatic beta cells in the islets of Langerhans, leading to loss of insulin secretion (1). The etiology of the rising incidence is not fully understood; the hygiene hypothesis links improved sanitary conditions to an increase in immune-mediated disorders (2). A recent meta-analysis of 126 studies from 55 countries estimated the global incidence at 14.07 per 100, 000 person-years, covering the periods before and during the COVID-19 pandemic (3), and an estimated 9.2 million people were living with T1D in 2024, including 1.8 million children and adolescents under 20 years of age (4). Genetic markers of T1D susceptibility are present from birth, whereas immunological markers (islet autoantibodies) and metabolic markers appear only after the onset of the autoimmune process and progressive beta-cell loss (5).

The strongest genetic association in T1D is with the human leukocyte antigen (HLA) class II locus, although multiple non-HLA loci also contribute to overall risk (6–9), including in patients lacking the classical HLA-DR3-DQ2 and HLA-DR4-DQ8 risk haplotypes (10, 11). The HLA class II region is estimated to account for approximately 41% of the familial aggregation of T1D (7). HLA class II molecules are heterodimers expressed on antigen-presenting cells, B lymphocytes, and the cells of the islets of Langerhans, where they present peptides to helper T lymphocytes (12). More than 90% of T1D patients carry the HLA-DRB1*03-DQB1*02 or HLA-DRB1*04-DQB1*03(DQ8) haplotype (13, 14), and comprehensive reviews and next-generation sequencing analyses continue to refine the catalogue of HLA alleles associated with T1D susceptibility and age at disease onset (15, 16). Recent mechanistic data show genotype-dependent differences in DNA methylation, with the HLA-DRB1*02-DQB1*06 haplotype exerting a protective effect while HLA-DRB1*03-DQB1*02 and HLA-DRB1*04-DQB1*03(DQ8) remain the principal risk haplotypes (17). In susceptible genotypes, HLA class II-mediated presentation of islet-derived peptides drives autoimmune destruction of beta cells (12).

Patients with T1D, especially children and adolescents, frequently develop additional autoimmune diseases (AIDs), most commonly autoimmune thyroiditis and coeliac disease (18). A large twin study has confirmed the shared genetic etiology of T1D and Hashimoto’s disease (19), and a recent British population-based analysis of 22 million individuals provides current data on the co-occurrence of various autoimmune diseases (20). Other autoimmune forms of diabetes such as latent autoimmune diabetes in adults (LADA) share HLA risk genotypes with T1D (21) and are addressed in current diagnostic classifications (22), whereas maturity-onset diabetes of the young (MODY), an autosomal-dominant monogenic disorder without autoantibodies, represents a distinct entity (23); contemporary guidelines and reviews describe the diagnostic and therapeutic approach to individual MODY subtypes (24, 25).

Autoimmune thyroid disease is particularly common among T1D patients, with up to 20% having circulating thyroid autoantibodies (anti-TPO or anti-Tg) (26) and a small proportion progressing to autoimmune hypothyroidism or, less commonly, hyperthyroidism (~1%) (27). Hashimoto’s thyroiditis is the leading cause of hypothyroidism in iodine-replete areas (28), and elevated anti-TPO antibodies correlate with lymphocytic infiltration of the thyroid (29). Annual screening with TSH and antithyroid antibodies is recommended for all children and adolescents with T1D (30), in line with current ADA and ISPAD guidelines (22). Specific HLA class II haplotypes contribute to thyroid autoimmunity risk in T1D, HLA-DRB1*03-DQB1*02/DRB1*04:01-DQB1*03:02 confers increased susceptibility, whereas HLA-DRB1*11:01-DQA1*05:05-DQB1*03:01 and HLA-DRB1*15:01-DQA1*01:02-DQB1*06:02 are associated with a protective effect (31), with additional evidence for shared HLA and non-HLA loci (PTPN22, CTLA-4, FOXP3) from twin (19) and gene-network studies (32).

Coeliac disease is an immune-mediated inflammatory disorder of the small intestine triggered by dietary gluten in genetically predisposed individuals and diagnosed according to updated criteria that include biopsy-free confirmation in selected paediatric cases (33). More than 99% of coeliac patients carry the HLA-DRB1*03-DQB1*02 and/or HLA-DRB1*04-DQB1*03(DQ8) haplotype (34), and approximately 5% of T1D patients develop coeliac disease, with most diagnoses established within five years of T1D onset (35). Recent national cohorts from Sweden and Poland confirm the high prevalence of coeliac disease in children with T1D (36, 37). Concurrent T1D-coeliac risk is highest for HLA-DR3-DQ2 and HLA-DR4-DQ8 haplotypes, whereas HLA-DQB1*06:02 and HLA-DRB1*04 subtypes may exert protective effects (38, 39), and specific DQ2.5/DQ8, DQ2.5/DQ2.5, and DQ2.5/DQ2.3 genotype combinations identify high-risk individuals in large cohorts (40). HLA class II variants are also central to broader autoimmune comorbidity patterns (41). Beyond T1D, coeliac disease is frequently associated with autoimmune thyroiditis, dermatitis herpetiformis, selective IgA deficiency (42), and Down and Turner syndromes (43).

Psoriasis is an immune-mediated inflammatory disease of the skin and joints, with the strongest HLA linkage at the HLA-C locus, notably HLA-C*06:02 and HLA-C*01 (44, 45); it shares the Th1/Th17 and TNF-α/IL-23/IL-17 pathways with T1D, and patients with psoriasis show an increased likelihood of co-occurring T1D, although the temporal direction of the association is not established (45). Atopic dermatitis is a chronic, relapsing inflammatory skin disease driven by a Th2-skewed immunopathology and epidermal barrier dysfunction; family history of atopy is the strongest known risk factor, and atopic dermatitis may co-occur with coeliac disease (46). Current joint AAAAI/ACAAI guidelines (47) and a Croatian multidisciplinary review (48) provide contemporary clinical management frameworks.

Although the association of the classical risk haplotypes HLA-DRB1*03-DQB1*02 and HLA-DRB1*04-DQB1*03(DQ8) with T1D is well established, data on specific HLA profiles in patients with T1D and concomitant AID, particularly in Central European populations, remain limited. Understanding these patterns may contribute to better risk stratification for autoimmune comorbidities, which is relevant for screening and early intervention, especially in the pediatric population in which disease onset predominates. The objective of this study was therefore to investigate the association of *HLA-DRB1*, -*DQA1*, and *-DQB1* allele variants, as well as *HLA-DQA1-DQB1-DRB1* haplotypes with T1D, and with T1D associated with concomitant AID affecting the thyroid, gastrointestinal tract, and skin.

## 2 Patients and methods

### 2.1 Study design

Cross-sectional study with historical data.

The study was conducted at the Department of Endocrinology, Clinic of Internal Medicine, and at the Department of HLA Typing and Genomic Diagnostics Clinical Institute of Transfusion Medicine, University Hospital Centre Osijek (UHCO), Republic of Croatia. HLA typing of patients was performed as part of routine clinical care between January 2009 and February 2026. Participants and controls.

A consecutive series of 149 unrelated patients referred for HLA typing for suspected T1D or as part of the diagnostic work-up between January 2009 and February 2026 were identified from the database of the Clinical Institute of Transfusion Medicine (CITM), UHCO. Inclusion criteria were: (i) clinically confirmed T1D according to ISPAD/ADA criteria (symptoms of hyperglycemia together with fasting plasma glucose ≥ 7.0 mmol/L, or 2-h OGTT plasma glucose ≥ 11.1 mmol/L, or HbA1c ≥ 6.5%, or positivity for at least one of the autoantibodies GAD-65, IA-2, IAA, or ZnT8); (ii) available HLA typing of the HLA-DRB1, -DQA1, and -DQB1 loci; (iii) available clinical data in the hospital information system (HIS) on concomitant autoimmune diseases. The sample size was based on all consecutive T1D patients typed at a single center during the 17-year period (convenience sample); a formal prospective power calculation was not performed.

Previously published allele and haplotype frequency data from 111 unrelated blood donors from eastern Croatia were used as the control (49). The control cohort comprised adult blood donors (median age 44 years, IQR 37-54; 79 men and 32 women) whose blood samples were collected between 2015 and 2017 from the same geographical region as the T1D participants. Controls were not individually matched to patients by age or sex, in line with the population-based reference cohort approach commonly applied in HLA association studies. HLA typing of the control cohort was performed at the Department of Pathological Physiology, Faculty of Medicine and Dentistry, Palacký University Olomouc, Czech Republic, using high-resolution commercial kits on an Illumina next-generation sequencing (NGS) platform. Both laboratories (the Department of HLA Typing and Genomic Diagnostics in Osijek and the Department of Pathological Physiology in Olomouc) participate in external quality assessment (EQA) schemes, ensuring the comparability of typing results. For statistical analysis, the high-resolution HLA typing results of the controls were converted to the corresponding low-resolution (allele groups) assignments according to the IPD-IMGT/HLA nomenclature (50).

### 2.2 Variables and diagnostic criteria

Main exposure: low resolution molecular typing of HLA-DRB1, -DQA1, and -DQB1 loci. Main outcome: the presence of at least one concomitant autoimmune disease diagnosed according to the following criteria: Hashimoto’s thyroiditis, positive anti-TPO and/or anti-Tg antibodies together with corresponding clinical or ultrasound findings (ICD-10 E06.3); Graves’ disease, positive TRAb antibodies together with hyperthyroidism (ICD-10 E05.0); coeliac disease, positive anti-tTG IgA antibodies and/or small-bowel biopsy with Marsh ≥ 2 (ICD-10 K90.0); atopic dermatitis, dermatological diagnosis based on the Hanifin-Rajka criteria (ICD-10 L20); psoriasis, dermatological diagnosis (ICD-10 L40).

#### 2.2.1 Methods

Clinical data (age, sex, diagnoses of concomitant AID) were extracted from the hospital information system (HIS) of UHCO using ICD-10 codes as the primary filter, supplemented by free-text review by trained medical personnel. HLA typing results were obtained from the database of the Clinical Institute of Transfusion Medicine, UHCO.

Genomic DNA was extracted from peripheral blood samples collected in EDTA tubes using a commercial extraction kit QIAamp DNA Blood Mini Kit (Qiagen, Hilden, Germany) according to the manufacturer’s instructions. DNA concentration and purity were verified spectrophotometrically. Low- to intermediate-resolution HLA typing of the HLA-DRB1, -DQA1, and -DQB1 loci was performed using PCR-based sequence specific oligonucleotide typing (PCR-SSOP/PCR-SSO). Commercialy available kits were used according to the manufacturer’s protocols, including Olerup SSP (CareDx, Stockholm, Sweden), HLA-FluoGene (inno-train Diagnostik, Kronberg, Germany), and Lifecodes (Immucor-Werfen, Barcelona, Spain). Allele nomenclature was aligned with the most recent IPD-IMGT/HLA Database releases at the time of typing. HLA typing was performed as part of routine clinical care between 2009 and 2026. Through the study period, the Department of HLA Typing and Genomic Diagnostics successfully participated in external quality assessment (EQA) schemes since 2009.

#### 2.2.2 Bias

The following potential sources of bias were considered in order to minimize their effect: (i) selection bias, the sample was derived from patients referred for HLA typing, which may limit generalizability since typing was not universal for all T1D diagnoses during the study period; (ii) detection bias, patients with a more complex clinical presentation (e.g., younger age at diagnosis, family history of autoimmune disease) may have been more frequently typed and more frequently investigated for concomitant autoimmune diseases, potentially overestimating the rate of comorbidities; furthermore, systematic screening for autoimmune thyroid disease (TSH, anti-TPO) and coeliac disease (tTG-IgA) in accordance with current ISPAD guidelines was not consistently applied throughout the study period, and diagnoses of concomitant autoimmune diseases relied primarily on clinical presentation and targeted investigations; (iii) classification bias, diagnoses of concomitant autoimmune diseases were extracted from the HIS, and undiagnosed (subclinical) cases cannot be excluded, leading to bias toward the null. Trained medical personnel extracted the data using standardized ICD-10 codes to harmonize classification.

### 2.3 Statistical methods

Data were analyzed using IBM SPSS Statistics version 29 (IBM Corp., Armonk, NY, USA), JASP version 0.19.5 (JASP Team, 2024), and GraphPad Prism version 11 (GraphPad Software, Boston, MA, USA). The HLA allele and haplotype frequencies were determined by direct counting. Probable haplotypes were inferred based on the established linkage disequilibrium between HLA-DQA1, -DQB1, and -DRB1 loci in individuals of European ancestry (51). The normality of continuous variables was assessed using the Shapiro-Wilk test. Categorical variables are presented as absolute and relative frequencies, whereas continuous data are presented as medians with interquartile ranges (IQR). Differences in continuous variables between two groups were assessed using the Mann-Whitney U test, whereas comparisons among more than two groups were performed using the Kruskal-Wallis test with Dunn’s *post hoc* test. Categorical variables were compared using Pearson’s chi-square test or Fisher’s exact test when expected cell counts were <5. For contingency tables containing zero cells, odds ratios and 95% confidence intervals were calculated using the Haldane-Anscombe correction. Statistical significance was set at α = 0.05, and all p-values were two-sided.

Population stratification was not formally assessed (e.g., by principal component analysis or genomic control), given that the study population was ethnically homogeneous, with all participants originating from north-eastern Croatia, a region characterized by low ethnic diversity and relatively uniform genetic background within the Central European (Slavic) cluster. Therefore, the risk of bias due to population stratification was considered low.

## 3 Results

### 3.1 Baseline characteristics of the participants

The study included 149 patients with diabetes and 111 healthy controls. Of the patients with diabetes, 94 (63.1%) had diabetes alone and 55 (36.9%) had one concomitant autoimmune disease. Patients with diabetes were significantly younger than healthy controls; 40 (26.8%) of the diabetes cohort were younger than 18 years of age. The concomitant conditions included autoimmune thyroid disease in 35 (23.5%) patients, autoimmune gastrointestinal disease in 11 (7.4%), and autoimmune skin disease in 9 (6.0%). The demographic and clinical characteristics of the study cohort are presented in **Table 1**.

**Table 1:** Demographic and clinical characteristics of the study cohort.

	Median (IQR) or N (%)			p*
	Control (N = 111)	Diabetes (N = 94)	Diabetes with AID (N = 55)	
Sex				
Male	79 (71.2)	55 (58.5)	19 (34.5)	<0.001^a^
Female	32 (28.8)	39 (41.5)	36 (65.5)	
Age (years)	44 (37 - 54)	21 (17 -26)	21 (18 – 26)	<0.001^b^
Age diabetes diagnosis (years)	–	9 (6 – 13)	10 (7 – 16)	0.15
Age AID diagnosis (years)			11 (7 – 15)	
Thyroiditis			24 (43.6)	
Hypothyroidism			11 (20.0)	
Celiac disease			8 (14.5)	
Irritable bowel syndrome			1 (1.8)	
Obesity			2 (3.6)	
Psoriasis			6 (10.9)	
Skin lupus erythematosus			1 (1.8)	
Pityriasis			1 (1.8)	
Atopic dermatitis			1 (1.8)	

AID, autoimmune disease; *Kruskal-Wallis test with Dunn’s *post hoc* test or Mann-Whitney U test for continuous variables, and Pearson’s chi-square test for categorical variables; a p < 0.05 for comparisons between the diabetes with AID group and both the control and diabetes groups; b p < 0.05 for comparisons between the control group and both diabetes groups.

### 3.2 Comparison of *HLA-DRB1*, *-DQA1*, and *-DQB1* allele variants distributions between groups of patients with T1D, T1D with concomitant AID, and control group

**Figure 1:**
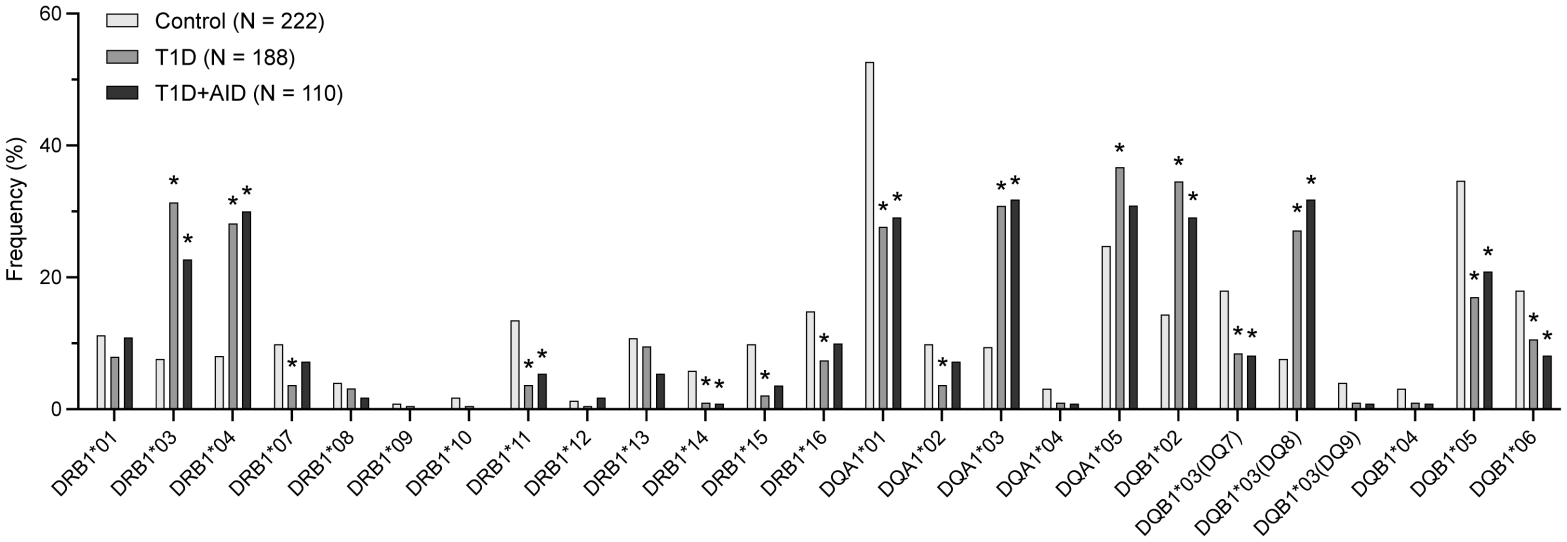
Comparison of HLA-*DRB1*, -*DQA1*, and -*DQB1* allele variants distributions between study groups. T1D, Type 1 diabetes mellitus; T1D+AID, T1D with concomitant autoimmune disease; *Fisher exact test, p < 0.05 compared to control.

Frequencies of *HLA-DRB1*, *-DQA1*, and *-DQB1* allele variants were analyzed in 188 alleles from patients with T1D alone, 110 alleles from patients with T1D with concomitant autoimmune disease, and 222 alleles from healthy controls. The HLA-DRB1*03, -DRB1*04, -DQA1*03, -DQB1*02 and -DQB1*03(DQ8) allele variants were significantly more frequent in both patient groups, T1D and T1D+AID, compared to healthy controls, whereas HLA-DQA1*05 was significantly more frequent in patients with T1D than in healthy controls. Conversely, HLA-DRB1*11, -DRB1*14, -DQA1*01, -DQB1*03(DQ7), -DQB1*05 and -DQB1*06 were significantly more frequent in healthy controls than in either patient group, whereas HLA-DRB1*07, -DRB1*15, -DRB1*16, and -DQA1*02 were significantly more frequent in healthy controls than in T1D group. No significant differences in allele variant frequencies were observed between T1D and T1D+AID groups. Complete allele frequency distributions and statistical comparisons are presented in **Figure 1**.

### 3.3 Comparison of *HLA-DQA1~DQB1~DRB1* haplotypes distributions between groups of patients with T1D, T1D with concomitant AID, and control group

**Figure 2:**
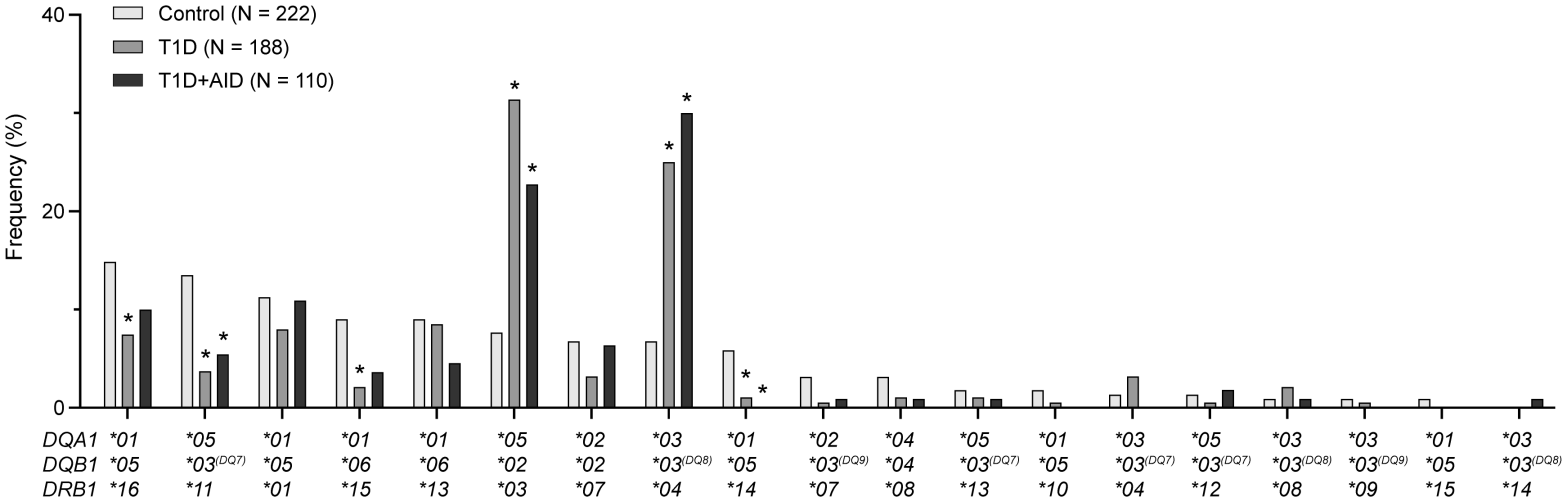
Comparison of *HLA-DQA1~DQB1~DRB1* haplotypes distributions between study groups. T1D, Type 1 diabetes mellitus; T1D+AID, T1D with concomitant autoimmune disease; *Fisher exact test, p < 0.05 compared to control.

No significant differences in the distribution of inferred haplotypes were observed between patients with T1D and T1D with concomitant AID. The HLA-DQA1*05~DQB1*02~DRB1*03 haplotype showed the greatest difference in frequency between patients with T1D and healthy controls (31.4% vs 7.7%, respectively). In patients with T1D and concomitant AID, the HLA-DQA1*03~DQB1*03(DQ8)~DRB1*04 haplotype exhibited the largest difference compared with healthy controls (30.0% vs 6.8%, respectively). The distributions of inferred haplotype frequencies across the study groups are shown in **Figure 2**.

### 3.4 Association of *HLA-DRB1*, *-DQA1*, and *-DQB1* allele variants with susceptibility to T1D, and T1D with concomitant AID

**Figure 3:**
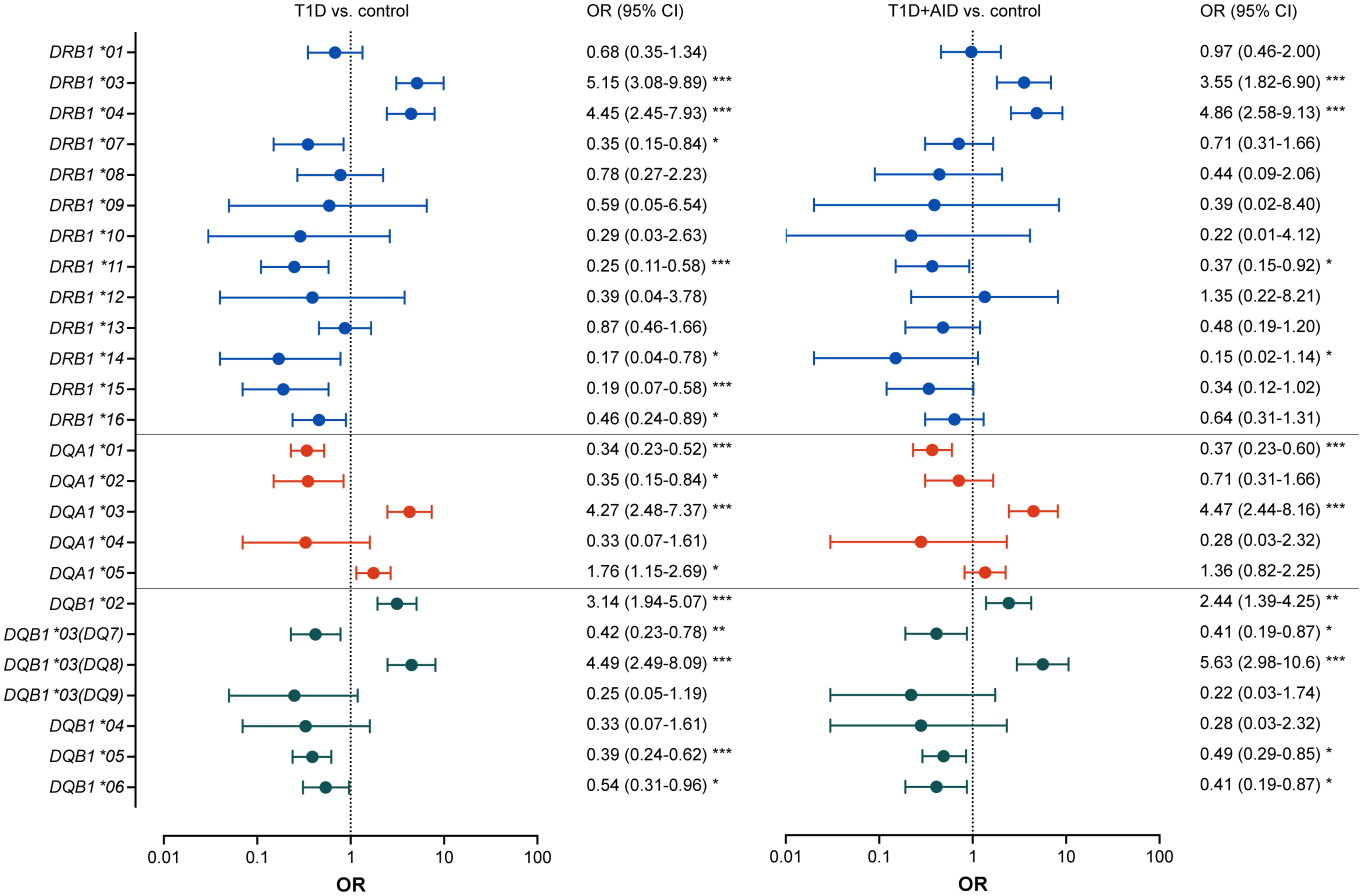
Association of inferred HLA-DRB1, -DQA1, and -DQB1 allele variants with the risk of T1D and T1D with concomitant AID. T1D, Type 1 diabetes mellitus; T1D+AID, T1D with concomitant autoimmune disease; OR, odds ratio; 95%CI, 95% confidence interval; ***Fisher exact test, p < 0.001, **p < 0.01, *p < 0.05.

Among the *HLA-DRB1* allele variants, HLA-DRB1*03 and -DRB1*04 were strongly associated with susceptibility to both, T1D and T1D with concomitant AID. HLA-DRB1*03 conferred the greatest risk for T1D (OR 5.15, 95%CI 3.08 - 9.89; p < 0.001), whereas HLA-DRB1*04 was associated with the highest risk of T1D and concomitant AID (OR 4.86, 95%CI 2.58 - 9.13; p < 0.001). Among the *HLA-DQA1* allele variants, HLA-DQA1*03 showed the strongest association with susceptibility to both T1D (OR 4.27, 95% CI 2.48 - 7.37; p < 0.001) and T1D with concomitant AID (OR 4.47, 95% CI 2.44 - 8.16, p < 0.001). Among the *HLA-DQB1* allele variants, HLA-DQB1*02 and -DQB1*03(DQ8) were associated with increased susceptibility to both T1D and T1D with concomitant AID. HLA-DQB1*03(DQ8) conferred the highest odds of disease in both patient groups (OR 4.49, 95%CI 2.49 - 8.09, p < 0.001 and OR 5.63, 95%CI 2.98 - 10.64; p < 0.001, respectively). In contrast, HLA-DRB1*14, -DRB1*11, and -DQB1*05 showed the strongest protective associations against T1D (OR 0.17, 95%CI 0.04 - 0.78, p = 0.02; OR 0.25, 95%CI 0.11 - 0.58, p < 0.001, and OR 0.36, 95%CI 0.24 - 0.62, p < 0.001, respectively). The odds ratios for T1D without and with concomitant AID according to *HLA-DRB1*, *-DQA1*, and *-DQB1* allelic variants are presented in **Figure 3**.

### 3.5 Association of *HLA-DQA1~DQB1~DRB1* haplotypes with the risk of T1D, and T1D with concomitant AID

**Figure 4:**
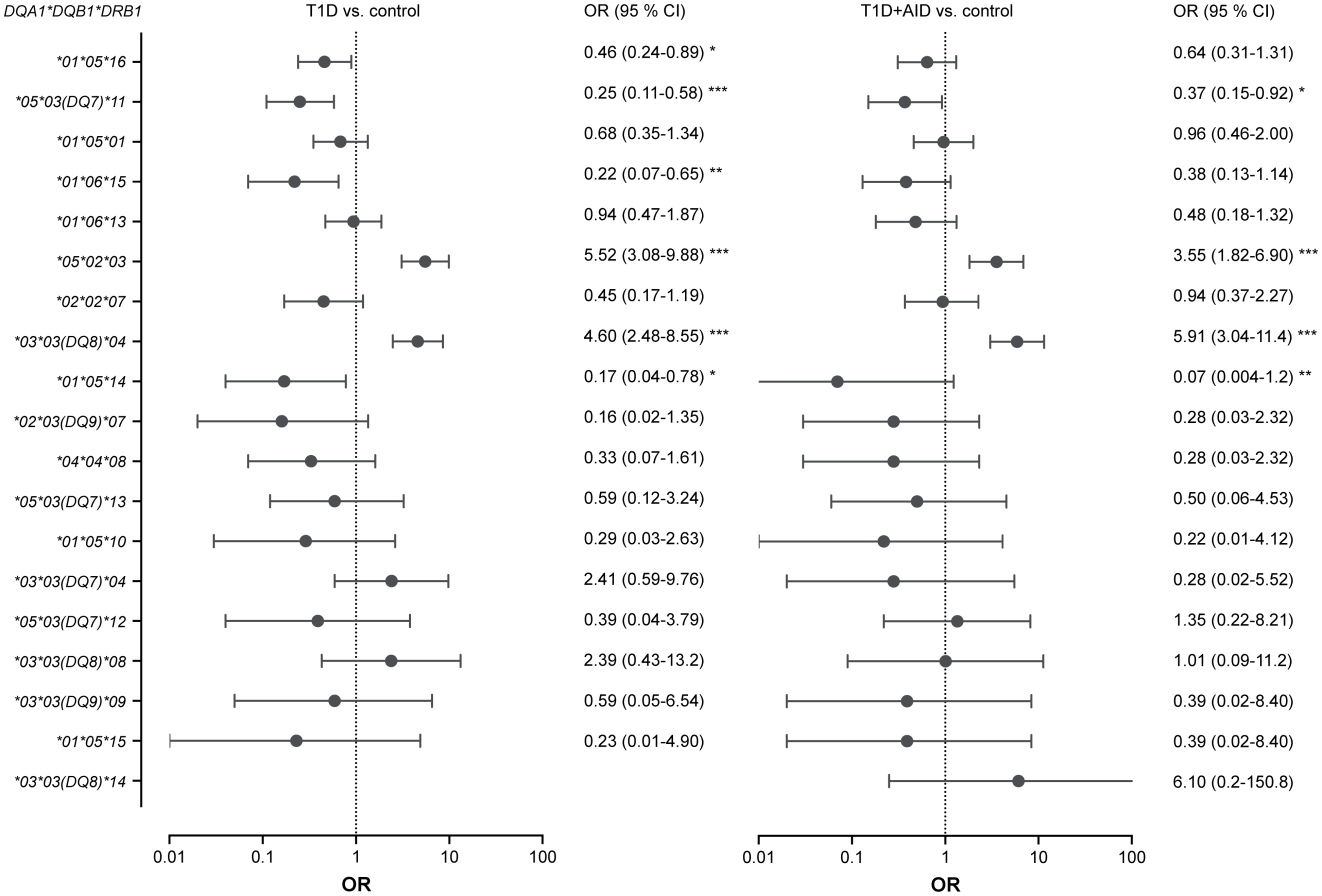
Association of inferred *HLA-DQA1~DQB1~DRB1* haplotypes with the risk of T1D and T1D with concomitant AID. T1D, Type 1 diabetes mellitus; T1D+AID, T1D with concomitant autoimmune disease; OR, odds ratio; 95%CI, 95% confidence interval; ***Fisher exact test, p < 0.001, **p < 0.01, *p < 0.05.

Among the inferred haplotypes, HLA-DQA1*05~DQB1*02~DRB1*03 and HLA-DQA1*03~DQB1*03(DQ8)~DRB1*04 emerged as the strongest susceptibility haplotypes for both T1D and T1D with concomitant AID. The HLA-DQA1*05~DQB1*02~DRB1*03 haplotype conferred the highest risk of T1D (OR 5.52, 95%CI 3.08 - 9.88; p < 0.001), whereas the HLA-DQA1*03~DQB1*03(DQ8)~DRB1*04 haplotype conferred the highest risk of T1D with concomitant AID (OR 5.91, 95%CI 3.04 - 11.49; p < 0.001). The odds ratios for T1D without and with concomitant autoimmune diseases according to inferred haplotypes are presented in **Figure 4**.

## 4 Discussion

Type 1 diabetes mellitus is a multifactorial disease caused by the interaction between genetic inheritance and environmental factors (8). The present study included 149 patients with an established HLA phenotype; the median age at T1D diagnosis was 9 years (IQR 6-13) in the T1D-alone group and 10 years (IQR 7-16) in patients with concomitant autoimmune disease, which is consistent with the study by Tattersall and colleagues, whose median was 11 years (23). The trend of decreasing age at diagnosis has been confirmed in a recent 50-year single-center analysis in which the median age at diagnosis decreased from 9.5 to 7.1 years over the follow-up period (52). Concomitant autoimmune diseases were recorded in 36.9% of our patients, which falls within the range of reported frequencies; the study by Warncke and colleagues reported a rate of 46.6% (18), and recent population-based studies confirm the co-occurrence of T1D with other autoimmune conditions, a twin study demonstrates a shared genetic etiology of T1D and Hashimoto’s disease (19), and a population study of 22 million inhabitants of the United Kingdom shows a significant co-occurrence of various autoimmune diseases, dependent on age, sex, and socioeconomic status (20).

In this study, the most common concomitant condition was autoimmune thyroid disease (23.5%), followed by autoimmune gastrointestinal disease (7.4%) and autoimmune skin disease (6.0%). These results correlate with the study by Warncke and colleagues, who recorded 19.6% autoimmune thyroid disease and 10.7% autoimmune gastrointestinal disease in T1D patients (18). The relatively lower frequency of skin autoimmune diseases in some prior reports may reflect differences in case ascertainment between cohorts and the potential under-recording of dermatological diagnoses in electronic health records.

Previous studies have identified HLA-DRB1, HLA-DQA1, and HLA-DQB1 as key genetic factors in the pathogenesis of T1D (6–9, 53, 54). In our cohort, HLA-DRB1*03 was the allele variant most strongly associated with T1D without concomitant autoimmune disease (OR 5.15, 95% CI 3.08 - 9.89; p < 0.001), whereas HLA-DRB1*04 was most strongly associated with T1D and concomitant autoimmune disease (OR 4.86, 95% CI 2.58 - 9.13; p < 0.001). At the haplotype level, the HLA-DQA1*05-DQB1*02-DRB1*03 (DR3-DQ2) haplotype showed the strongest association with T1D alone (OR 5.52, 95% CI 3.08 - 9.88; p < 0.001), whereas the HLA-DQA1*03-DQB1*03(DQ8)-DRB1*04 (DR4-DQ8) haplotype showed the strongest association with T1D and concomitant autoimmune disease (OR 5.91, 95% CI 3.04 - 11.49; p < 0.001). Importantly, however, direct comparison between the T1D-alone and T1D+AID groups did not reveal any significant differences in allele or haplotype frequencies. This pattern indicates that DR3-DQ2 and DR4-DQ8 are both risk haplotypes for T1D and for T1D with concomitant autoimmune disease when each is compared with the healthy control population, but the present study does not provide statistical support for a differential predisposition of DR4-DQ8 towards the combined autoimmune phenotype relative to T1D alone. Rowe and colleagues similarly identified HLA-DQB1*03:02 (DQ8) and HLA-DQB1*02:01 as the most common alleles in patients with T1D and concomitant autoimmune diseases (13). The prevalence of high-risk genotypes is higher in North American and European populations, and approximately 5% of children carrying the HLA-DQB1*03:02/HLA-DQB1*02:01 genotype develop T1D earlier than children with other genotypes (13). Patients with T1D are at increased risk of having other autoimmune diseases, most commonly autoimmune thyroiditis and coeliac disease (55).

Among the allele- and haplotype-level comparisons presented in **Figures 3**, **4**, the nominally significant protective association of HLA-DRB1*14 allele and the HLA-DQA1*01~DQB1*05~DRB1*14 haplotype in patients with T1D and concomitant autoimmune disease should be interpreted with caution. The upper bounds of the 95% confidence interval for the corresponding odds ratios exceeded 1, indicating that the estimate does not exclude the null value with sufficient precision. This finding most likely reflects the small number of carriers in this subgroup and the resulting instability of the odds ratio estimate, further accentuated by the use of the Haldane-Anscombe correction for sparse data. Accordingly, the observed association with HLA-DRB1*14 should be considered exploratory and hypothesis-generating until confirmed in larger, adequately powered cohorts.

In contrast, the protective associations identified in our cohort were internally consistent and biologically plausible, with HLA-DRB1*15, -DRB1*11, and -DQB1*05 all being significantly under-represented in patients with T1D. This pattern is consistent with the well-established protective effect of the HLA-DRB1*15:01-DQA1*01:02-DQB1*06:02 haplotype, which represents the strongest protective HLA class II association in T1D (15, 17), as well as with previous reports demonstrating a protective role of the HLA-DRB1*11:01 allele and HLA-DRB1*15:01-DQB1*06:02 haplotype against thyroid autoimmunity (31). Because HLA typing was performed at low- to intermediate-resolution, specific protective alleles, most importantly DQB1*06:02, could not be directly resolved. Additionaly, the observed HLA-DRB1*11 and HLA-DQB1*05 associations are more likely to reflect their occurrence within protective HLA haplotypes than independent protective effects of these allele groups (16).

### 4.1 Autoimmune thyroid disease

Among patients with T1D and concomitant autoimmune disease in our cohort, autoimmune thyroid disease was the most common manifestation. Flesch and colleagues showed that the HLA-DQB1*03:02 and HLA-DRB1*04:01 alleles are associated with the risk of developing autoimmune thyroid disease in T1D patients (31). A contemporary review of shared HLA and non-HLA loci (PTPN22, CTLA-4, FOXP3) in T1D and AITD (32), and a recent mechanistic study of DNA methylation according to risk/protective genotypes (17), confirm and complement these findings. Our observation that HLA-DRB1*04 and the HLA-DQA1*03-DQB1*03(DQ8)-DRB1*04 (DR4-DQ8) haplotype were most strongly associated with T1D and concomitant autoimmune disease is consistent with the recognized role of the DR4-DQ8 haplotype in T1D susceptibility and in T1D-associated autoimmune phenotypes; however, because a direct comparison between the T1D-alone and T1D+AID groups did not reveal significant differences, this haplotype should be interpreted as increasing the risk of both phenotypes relative to healthy controls rather than as specifically differentiating patients with autoimmune comorbidities. Approximately one-quarter of T1D patients develop autoimmune thyroid disease (55); about 20% have positive antithyroid antibodies, and 2 to 5% develop autoimmune hypothyroidism (26). Hyperthyroidism is rarer (~1%), but more common than in the general population (27).

### 4.2 Autoimmune gastrointestinal disease

The principal autoimmune gastrointestinal condition in T1D patients is coeliac disease, which shares the HLA-DR3-DQ2 and HLA-DR4-DQ8 susceptibility background with T1D itself. Our observation that HLA-DRB1*04 and the DR4-DQ8 haplotype were most strongly associated with T1D and concomitant autoimmune disease is consistent with the recognized overlap of HLA susceptibility between T1D and coeliac disease. Kozhakhmetova and colleagues reported a higher occurrence of the HLA-DRB1*04 allele in T1D patients with co-existing non-islet autoimmunity (56), which aligns with our findings. A recent large Sardinian cohort of 1, 886 patients identified the HLA-DQ2.5/DQ8, DQ2.5/DQ2.5, and DQ2.5/DQ2.3 genotypes as high-risk for the concurrent occurrence of coeliac disease and T1D (40). Approximately one-tenth of patients with T1D and coeliac disease have autoantibodies against transglutaminase, and half of them have high antibody levels and histologically confirmed coeliac disease (57). Approximately 5% of T1D patients develop coeliac disease (58), with the diagnosis usually made within five years of T1D onset (35). The clinical picture often includes poor glycemic control, recurrent hypoglycemic episodes, unpredictable glucose measurements, and growth retardation due to malabsorption (59).

### 4.3 Autoimmune skin disease

In our cohort, autoimmune skin disease (comprising psoriasis and atopic dermatitis) was the least common concomitant condition (6.0%). Patients with psoriasis have an increased likelihood of co-occurring T1D, although the temporal direction of this association remains unclear; the Th1/Th17 axis and the cytokines TNF-α, IL-23, and IL-17 are central to the pathogenesis of both diseases (45). Ogawa and Okada describe an increased occurrence of psoriasis in genetically predisposed individuals of European populations carrying polymorphisms of HLA-DQA, and of HLA-DQB in the Japanese population, suggesting a role for structural changes in antigen presentation (60). Although earlier studies have linked specific HLA-DR and HLA-DQ variants with atopic dermatitis, the findings to date are inconsistent and depend on the ethnic background of the studied population; current guidelines and review articles focus on the immunopathogenesis mediated by the Th2 axis and epidermal barrier dysfunction, while the role of HLA genetics continues to be intensively investigated (47, 48). Notably, vitiligo, an autoimmune skin condition that shares the HLA-DR3 and HLA-DR4 background with T1D and is generally regarded as biologically more closely related to T1D than psoriasis or atopic dermatitis, was not identified in our cohort. This absence most likely reflects a selection artefact: dermatological diagnoses were not systematically screened for in the participating patients, and vitiligo may be under-recorded in the hospital information system compared with conditions with a more prominent inflammatory or immunological work-up. Given the small number of patients with autoimmune skin disease in our cohort (n = 9), targeted HLA–skin disease associations could not be evaluated separately, and the HLA profile observed in this subgroup should be interpreted as reflecting the general T1D risk pattern rather than skin-specific associations.

The present study examined the frequency of individual HLA alleles and haplotypes in patients with T1D and concomitant autoimmune diseases. Given the increasing occurrence of T1D, similar studies should include a larger number of participants; because of the complexity of the HLA system and its inheritance, there is a need for further research to enable successful population screening and to achieve early diagnosis, early treatment, and prevention in families with cases of T1D and concomitant autoimmune diseases.

## 5 Study limitations

This study has several limitations that should be considered when interpreting the results. First, it is a single-center cross-sectional study, which precludes causal inference and prediction of future autoimmune comorbidity development; prospective longitudinal cohorts would be required to determine whether specific HLA haplotypes predict the onset of additional autoimmune diseases in patients with T1D. Second, low- to intermediate-resolution HLA typing does not distinguish between individual allele subtypes (e.g., HLA-DRB1*04:01 versus HLA-DRB1*04:05) that differ in the degree of association with T1D. Although patient and control HLA typing were performed using different platforms (PCR-SSOP versus NGS), both approaches are validated for HLA-DRB1, -DQA1, and -DQB1 typing, results from both laboratories are subject to external quality assessment, and high-resolution control data were harmonized to low-resolution equivalents according to IPD-IMGT/HLA nomenclature, minimizing methodological bias. Third, the number of patients in the subgroups with individual comorbidities (particularly autoimmune skin diseases, n = 9) limits statistical power and may inflate odds ratio estimates in small cells. Fourth, patients with autoimmune comorbidities were pooled into a single T1D+AID group, which reflects a methodological compromise imposed by the small disease-specific subgroup sizes. Because Hashimoto’s thyroiditis, Graves’ disease, coeliac disease, psoriasis, and atopic dermatitis are known to have partially overlapping but partially distinct HLA class II susceptibility profiles, pooling may dilute disease-specific signals and could obscure opposing associations (protective for one condition, predisposing for another) that cancel out when combined. Fifth, systematic screening for autoimmune thyroid disease (TSH, anti-TPO) and coeliac disease (tTG-IgA) in line with current ISPAD recommendations was not consistently performed throughout the study period; consequently, subclinical or asymptomatic cases may have remained undiagnosed, leading to detection bias and probable underestimation of the true prevalence of concomitant autoimmune diseases in this cohort. Sixth, owing to the retrospective extraction of data from the HIS, additional diagnoses that were not actively investigated may have been missed; in particular, vitiligo, which shares HLA-DR3 and HLA-DR4 background with T1D and is biologically more closely related to T1D than psoriasis or atopic dermatitis, may be under-ascertained in dermatological records and was not identified in our cohort. Seventh, the control cohort was drawn from adult blood donors (median age 44 years), and was therefore substantially older than the patient groups (median age 21 years); although HLA allele frequencies are generally considered stable across adult age strata within the same ethnic population, this age difference implies that some controls may harbour latent, undiagnosed autoimmune conditions, which could bias the prevalence comparison. Eighth, the study population is ethnically homogeneous (Central European), and generalization of the findings to other ethnic groups requires independent replication.

## 6 Conclusion

In this single-center Central European cohort of 149 patients with type 1 diabetes mellitus, a concomitant autoimmune disease was present in 36.9% of patients, most commonly autoimmune thyroid disease (23.5%), followed by autoimmune gastrointestinal disease (7.4%) and autoimmune skin disease (6.0%). Both patient groups showed a marked enrichment of the classical HLA class II risk profile compared with healthy controls. The HLA-DRB1*03 allele variant and the HLA-DQA1*05-DQB1*02-DRB1*03 (DR3-DQ2) haplotype showed the strongest association with T1D without concomitant autoimmunity (OR 5.15, 95% CI 3.08-9.89; and OR 5.52, 95% CI 3.08-9.88, respectively; both p < 0.001), whereas HLA-DRB1*04 and the HLA-DQA1*03-DQB1*03(DQ8)-DRB1*04 (DR4-DQ8) haplotype showed the strongest association with T1D and concomitant autoimmune disease (OR 4.86, 95% CI 2.58-9.13; and OR 5.91, 95% CI 3.04-11.49, respectively; both p < 0.001). HLA-DQA1*03 and HLA-DQB1*03(DQ8) were significantly more frequent in both patient groups than in healthy controls, whereas no significant differences in allele or haplotype distribution were observed directly between patients with T1D alone and those with concomitant autoimmune disease. These findings confirm the well-established role of the HLA-DR3-DQ2 and HLA-DR4-DQ8 haplotypes in genetic susceptibility to T1D in a Central European population. Because a direct comparison between the T1D-alone and T1D with concomitant autoimmune disease groups did not reveal significant differences, DR4-DQ8 should be interpreted as being associated with an increased risk of both phenotypes relative to healthy controls, rather than as specifically distinguishing patients with autoimmune comorbidities from those without. Owing to the small number of patients within individual comorbidity subgroups, particularly autoimmune skin disease (n = 9), disease-specific HLA associations could not be evaluated separately; the HLA profile observed in these subgroups should therefore be regarded as reflecting the general T1D risk pattern rather than disease-specific associations. Because the study is cross-sectional, the observed HLA associations should be interpreted as correlations rather than predictors of future autoimmune disease development. Independent replication in larger, multicenter, prospective cohorts with high-resolution HLA typing is needed before these findings can be applied in clinical practice.

## Data Availability

The original contributions presented in the study are included in the article/supplementary material. Further inquiries can be directed to the corresponding authors.
